# TaMYB29: A Novel R2R3-MYB Transcription Factor Involved in Wheat Defense Against Stripe Rust

**DOI:** 10.3389/fpls.2021.783388

**Published:** 2021-11-29

**Authors:** Xiaoxu Zhu, Xiang Li, Qi He, Dongxiao Guo, Caiqi Liu, Junying Cao, Zhongyi Wu, Zhensheng Kang, Xiaojing Wang

**Affiliations:** ^1^State Key Laboratory of Crop Stress Biology for Arid Areas, College of Life Sciences, Northwest A&F University, Yangling, China; ^2^State Key Laboratory of Crop Stress Biology for Arid Areas, College of Plant Protection, Northwest A&F University, Yangling, China; ^3^State Key Laboratory of Crop Stress Biology for Arid Areas, College of Innovation and Experiment, Northwest A&F University, Yangling, China

**Keywords:** TaMYB29, transcription factor, reactive oxygen species, hypersensitive response, stripe rust, VIGS

## Abstract

Members of the R2R3-MYB transcription factor superfamily have been implicated in plant development, improved disease resistance, and defense responses to several types of stresses. To study the function of TaMYB29 transcription factor—a member of the R2R3-MYB superfamily—in response to an avirulent race of stripe rust pathogen, *Puccinia striiformis* f. sp. *tritici* (*Pst*), we identified and cloned the *TaMYB29* gene from wheat cultivar (cv.) AvS+*Yr10* following infection with *Pst*. The TaMYB29 protein, comprising 261 amino acids, contains two highly conserved MYB domains. We first showed that TaMYB29 is a transcription factor, whose transcriptional levels are significantly induced by salicylic acid (SA), abscisic acid (ABA), jasmonic acid (JA), ethylene (ET), and *Pst*. The results showed that TaMYB29 is involved in the wheat response to stipe rust. The overexpression of the *TaMYB29* gene resulted in the accumulation of reactive oxygen species (ROS) and pathogen-independent cell death in *Nicotiana benthamiana* leaves. The silencing of *TaMYB29* gene in wheat cv. AvS+*Yr10*, containing the stripe rust resistance gene *Yr10*, promoted hyphae growth, significantly downregulated the expression of pathogenesis-related (*PR*) genes, and substantially reduced the wheat resistance to *Pst* compared with the non-silenced control. In addition, the accumulation of hydrogen peroxide (H_2_O_2_) significantly decreased, and the activity of catalase, an enzyme required for H_2_O_2_ scavenging, was elevated. Altogether, TaMYB29 positively regulates the defense response against stripe rust in wheat AvS+*Yr10* by enhancing H_2_O_2_ accumulation, *PR* gene expression, and SA signaling pathway-induced cell death. These results provide new insights into the contribution of TaMYB29 to the defense response against rust pathogens in wheat.

## Introduction

Wheat is an important food crop grown worldwide and the second most consumed crop in China (Tian et al., 2012). *Puccinia striiformis* f. sp. *tritici* (*Pst*), a fungal pathogen and causative agent of stripe rust disease, has severely affected the annual yield of wheat (Park et al., 2007; Chen et al., 2014). Several control measures have been implemented worldwide to control this pathogen; these aim at enhancing the tolerance of wheat to the infection (Seo and Park, 2010; Zhang et al., 2012a). The members of the Myeloblastosis (*MYB*) gene superfamily have been reported to actively participate in the developmental processes and defense responses of plants, attracting the worldwide attention of several plant scientists (Fujita et al., 2006; Wang et al., 2015).

Being one of the largest superfamilies of transcription factors in plants, several *MYB* genes have been isolated and identified from different plants (Dubos et al., 2010). The MYB protein contains a protein domain, typically 50 to 53-amino acid long, located near its N-terminus. MYB protein is divided into four categories, namely, 4R-MYB, R1R2R3-MYB, R2R3-MYB, and MYB-related, based on the number of repeats in this domain (Li et al., 2017; Zhang et al., 2018). The majority of MYB proteins belong to the R2R3-MYB gene subfamily. The R2R3-MYB proteins contain three uniformly spaced tryptophan residues within the MYB domain regions, except for R3, in which the first tryptophan residue is replaced by phenylalanine. The MYB repeat has a helix-turn-helix conformation, which forms a hydrophobic core and binds to DNA major grooves to exert their effects during transcription (Ogata and Nishimura, 1995). The first plant *MYB* gene *COLORED1* was cloned and identified from maize (*Zea mays*) and is involved in anthocyanin biosynthesis (Paz-Ares et al., 1987). Since then, functions of several *R2R3-MYB* genes have been discovered and reported in different plant species, including fundamental metabolism, plant growth, cell apoptosis, defense response to abiotic and biotic stresses, and signal transduction (Kranz et al., 1998; Cedroni et al., 2003; Zheng et al., 2017).

The *MYB* gene supports a wide range of signaling crosstalks between biotic and abiotic stress signals (Grant et al., 2003; Chini et al., 2010). For example, the AtMYB96 transcription factor is involved in abscisic acid (ABA)-mediated drought response. It enhances the pathogen resistance of plants by promoting the biosynthesis of salicylic acid (SA), implying that the *MYB* gene affects the ABA–SA signaling crosstalk (Seo and Park, 2010). Similarly, *AtMYB102* responds to salt stress, JA, ABA, wounds, and defense against herbivore attacks (De Vos et al., 2006). *AtMYB108* is involved in biotic and abiotic stress crosstalks and regulated by JA (Mengiste et al., 2003). *AtMYB15* is known to improve drought and salt tolerance in *Arabidopsis*, possibly by the ABA signaling pathway (Ding et al., 2009). TaPIMP1, an R2R3 MYB transcription factor in wheat, positively modulates defense responses to *Bipolaris sorokiniana* and drought stress *via* the ABA–SA signaling pathways in wheat (Zhang et al., 2012b). These findings indicate that several *MYB* genes execute their functions in a highly coordinated manner through a complex signaling network.

At the level of plant–pathogen interaction, a lot of previous studies demonstrated that the transcriptional products of *MYB* genes regulate plant disease resistance. In this regard, *AtMYB30* is identified as a positive regulator during a hypersensitive response (HR) to pathogen attacks in plants (Vailleau et al., 2002; Raffaele et al., 2006). *AtMYB46* modulates the disease susceptibility of *Arabidopsis* to the fungal pathogen *Botrytis cinerea* (Ramirez et al., 2011). Similarly, *AtMYB73* of *Arabidopsis thaliana* is implicated in *NPR1*-mediated SA and JA signaling pathways against necrotrophic fungal pathogen *Bipolaris oryzae* (Jia et al., 2011). *AtMYB108* confers plant resistance to the necrotrophic fungal pathogen *Alternaria brassicicola* (Mengiste et al., 2003). The tobacco *MYB1* gene induced by *Tobacco mosaic virus* infection participates in HR reactions and systemic acquired resistance (SAR). It is located downstream of the SA regulatory pathway and regulates the expression of *PR1* and other disease-related genes (Yang and Klessig, 1996). The overexpression of R2R3-MYB gene *TiMYB2R*-1 from *Thinopyrum intermedium* is known to significantly improve the resistance of transgenic wheat to the take-all disease (Liu et al., 2013). The barley MYB transcription factor gene *HvMYB6* confers resistance in barley to powdery mildew. Silencing of this gene increases the sensitivity of barley to *Blumeria graminis*, the causative agent of powdery mildew, whereas its overexpression enhances the disease resistance of transgenic barley (Chang et al., 2013). In wheat, the overexpression of *TaMYB86* significantly enhances the root rot resistance of transgenic wheat (Shan et al., 2016). *TaMYB4* shows a high similarity to certain R2R3-MYB transcription factors and is implicated in the signaling pathways activated by ABA, ET, and SA hormones to induce stress defense response. In addition, it can promote programmed cell death and increase wheat tolerance to *Pst* in the infection phase (Ma et al., 2011; Al-Attala et al., 2014). Overexpression and underexpression experiments of the wheat R2R3 *MYB* gene *TaPIMP1* show that *TaPIMP1* provides defense against *B. sorokiniana* (Zhang et al., 2012b).

Although several MYB transcription factors in wheat have been functionally studied, more in-depth studies are needed to reveal the function of the *MYB* genes in the complex hexaploid wheat. Zhang et al. (2012a) isolated 60 wheat *MYB* genes containing full-length gene sequences, of which 20 genes were reported to participate in defense response to multiple abiotic stresses involving complicated signaling pathways. Although MYB is the largest transcription factor family in plants, the literature on its functions related to wheat disease resistance is scarce. To elucidate the molecular regulatory mechanisms involved in wheat–*Pst* interaction, we isolated a highly induced *MYB* gene from wheat cultivar (cv.) AvS+*Yr10* after inoculating it with stripe rust fungus. The gene showed a sequence similar to that of the Chinese Spring *TaMYB29*. The *TaMYB29* gene was induced by high salt and exogenous ABA (Zhang et al., 2012a). In addition, *TaMYB29* along with *TaMYB19* and *TaMYB44* can co-regulate wheat plant phloem-based defense (PBD) against phloem-feeding insects; this function is executed through crosstalk with the ET signaling pathway (Zhai et al., 2017). These results imply the ability of *TaMYB29* to respond to biotic and abiotic stresses and suggest that its function is mediated *via* different plant signaling pathways. Therefore, we further wanted to characterize the function of TaMYB29 during the wheat defense response against stripe rust. In this study, we characterized three *TaMYB29* homologous genes, *TaMYB29-5A*, *TaMYB29-5B*, and *TaMYB29-5D* from wheat cv. AvS+*Yr10* and investigated the subcellular localization and transcription activation properties of the TaMYB29-5B protein. The transcript abundance of *TaMYB29* was studied using quantitative real-time PCR (qRT-PCR) in AvS and its near-isogenic lines (NILs) AvS+*Yr10* seedlings inoculated with *Pst* pathotype CYR32, and in response to different exogenous hormone treatments. The transient expression of *TaMYB29-5B* confers auto active HR response to tobacco. Furthermore, we used barley stripe mosaic virus-induced gene silencing (BSMV-VIGS) to show that *TaMYB29* is involved in defense against *Pst*. The relationships between *TaMYB29* silencing and SA concentration, accumulation of reactive oxygen species (ROS), and the expression of pathogenesis-related (*PR*) genes were studied. In addition, we evaluated the pathogen growth and cell death. Our results suggest that TaMYB29 is a positive regulator of resistance to *Pst* in wheat and executes this function through the accumulation of SA and ROS. We believe that the results of this study contribute to an increased understanding of the structure and function of TaMYB29.

## Materials and Methods

### Plant Material

Two wheat (*Triticum aestivum* L.) cultivars, tobacco (*Nicotiana benthamiana*), and *Pst* pathotype CYR32 were used in this study. Wheat AvocetS (AvS) and its near-isogenic line AvS*Yr10*NIL (AvS+*Yr10*) wheat seedlings carrying the *Yr10* resistance gene were grown in a growth chamber at 14°C under a 16h light/8h dark photoperiod. *N. benthamiana* was maintained in an artificial climate room with a temperature range of 20 to 22°C, a light intensity of 20,000Lux, and a 16-h photoperiod.

### Pathogen and Inoculation

The *Pst* race CYR32 that is compatible to wheat AvS and incompatible to AvS+*Yr10* was provided by the State Key Laboratory of Crop Stress Biology for Arid Areas, NWAFU, China. The uredospores of the CYR32 pathotype were suspended in isohexadecane (IHD) and sprayed evenly on the first leaves of AvS and AvS+*Yr10* wheat seedlings using a small air pump. After inoculation, these seedlings were maintained for 24h in a dark chamber with 100% relative humidity. These were subsequently returned to the original photoperiod condition. The seedlings in the control group were treated similarly with IHD containing no uredospores.

### RNA Extraction From Wheat Leaves and cDNA Preparation

The wheat leaves were sampled at 0, 12, 24, 48, 72, and 120h post-inoculation (hpi) and immediately frozen in liquid nitrogen and stored at −80°C before extraction of total RNA. The total RNA of wheat leaves was extracted using the Qiagen RNeasy Plant Mini Kit (Qiagen, Valencia, CA, United States) following the manufacturer’s instructions. The quality and concentration of RNA were assessed using a 1.5% agarose gel electrophoresis and 260/280_abs_ measurement using a NanoDrop 2000 spectrophotometer (Thermo Fisher Scientific, United States), respectively. Next, cDNAs were synthesized using the RevertAid First Strand cDNA Synthesis Kit (Thermo Fisher Scientific, United States) following the manufacturer’s protocol.

### Cloning and Sequence Analysis of *TaMYB29* Gene

To clone the *TaMYB29* gene, a pair of primers (M29-F/R) were designed using the Primer 5.0 software. The primers were designed based on the *TaMYB29* sequence released by the Chinese Academy of Agricultural Sciences in 2012 (NCBI GenBank Accession No. JF951912.1); these were largely used to amplify the open reading frame (ORF) of the *TaMYB29* gene. The PCR program was as follows: 3min at 95°C, followed by 35cycles of 30s at 95°C, 30s at 55°C, and 60s at 72°C, and finally 10min at 72°C. The PCR mixture contained the following: 0.125μl of Takara Ex Taq, 2.5μl of 10× Ex Tag buffer, 2μl of dNTP mixture, 2μl of template, and 1μl of each primer. Next, the total volume was made up to 20μl with ddH_2_O. The target fragments were inserted into the pGEM-T Easy vector (Promega, Madison, WI, United States) and sequenced. The *TaMYB29* sequence was used to BLAST the related MYB genes at the National Center for Biotechnology Information^1^ and the Ensemble plant database at http://plants.ensembl.org/index.html. The DNAman program (Lynnon Biosoft, Quebec, Canada) was used to align all nucleotide sequences of these genes and corresponding deduced protein sequences.

### RNA Analysis Using Quantitative Real-Time PCR

To determine the expression profiles of *TaMYB29* after different treatments, a pair of primers (M29-qRT-F/R) were designed. In addition, four pairs of primers were designed to detect the expression patterns of *TaPR1*, *TaPR2*, *TaPR5*, and *TaCAT* genes by quantitative real-time PCR (qRT-PCR). Wheat housekeeping gene *actin* was used as an internal control. All primers used are listed in Supplementary Table S1. The qRT-PCRs were performed on a CFX96 Real-Time System (Bio-Rad, Munich, Germany). The reactions were performed in a 20μl volume containing 10μl of 2×UltraSYBR mixture, 2μl of diluted cDNA, 1μl of each primer, and 6μl of ddH_2_O. The amplification conditions were as follows: a pre-denaturation for 10min at 95°C, followed by 40cycles of 15s at 95°C and 60s at 60°C. A dissociation curve was generated for every reaction to ensure specific amplification. Each reaction was performed in triplicate, and reactions without templates were used as negative controls. Three independent biological replicates were used for each time point and treatment. The 2^−ΔΔCt^ method was applied to analyze the experimental results (Livak and Schmittgen, 2001).

### Determination of Endogenous SA Concentration in Wheat Leaves

To measure the endogenous SA concentration under both compatible and incompatible interactions, the wheat leaves were sampled at 0, 12, 24, 48, 72, and 120hpi with CYR32 pathotype. These were frozen in liquid nitrogen immediately. The SA concentration was analyzed using liquid chromatography-mass spectrometry (LC-MS) on an LC-30A+TripleTOF5600+ machine (AB Sciex, Singapore) according to Wang et al.’s (2017) method. In brief, 200mg of the sample was ground into a powder in liquid nitrogen and subsequently extracted with 750μl of MeOH–H_2_O–HOAc (90:9:1, v/v/v). The solution was centrifuged at a high speed of 10,000rpm. Next, the supernatant was collected and dried under nitrogen. The extract was dissolved in 1,000μl of HPLC-grade MeOH and filtered using a Millex-HV 0.22μm filter (Millipore, Bedford, United States). Three independent biological replicates were used for each time point.

### Expression Profiles of *TaMYB29* Under Hormone Treatments

To analyze the expression profiles of TaMYB29 under different hormone treatments, the one-leaf stage wheat seedlings of AvS+*Yr10* were sprayed with 2μm methyl jasmonate (MeJA), 2μm ethylene (ET), 2μm abscisic acid (ABA), and 20μm salicylic acid (SA), following Wang et al.’s (2013) method. The mock control seedlings were similarly treated with an equal volume of distilled water. Subsequently, the seedlings were cut with a sterilized scissor for sampling at 0h, 2h, 6h, 12h, 24h, and 48h post-treatment (hpt). The samples were immediately frozen in liquid nitrogen and stored at −80°C. Three independent biological replicates were used for each time point and control.

### Subcellular Localization of TaMYB29 Protein

To study the subcellular localization of TaMYB29 protein, a pair of primers (163-M29-F/R) with restriction enzyme *Hin*dIII and *Bam*HI sites were designed. The PCR product of *TaMYB29* was subcloned into the 16318GFP vector. Next, the recombinant plasmid was amplified in *Escherichia coli* strain DH5α and extracted using the OMEGA Plasmid Maxi Kit. The wheat leaves were processed with cellulase R10 (Yakult Honsha) and macerozyme R10 (Yakult Honsha) to isolate protoplasts as previously described (Yoo et al., 2007). The TaMYB29-eGFP fusion vector and only eGFP vector (control) were transformed into the wheat protoplasts separately using PEG4000. The treated wheat protoplasts were maintained in the dark at 23°C for 24h and subsequently observed under a fluorescence microscope (Olympus Corporation, Tokyo, Japan).

In addition, the gateway cloning technology was used to link the *TaMYB29* gene to the pK7WGF2.0 vector carrying an enhanced green fluorescent protein (eGFP) tag. The pK7-TaMYB29-eGFP and pK7-eGFP vectors served as controls were transformed into the *Agrobacterium* strain GV3101 (pSoup-p19) by electroporation. Strains carrying different recombinant vectors were cultured to an optical density of 0.8 at 600nm (OD600) and injected into 4-week-old *N. benthamiana* leaves using a 1.0ml syringe without a needle. The transformed *N. benthamiana* were maintained in a greenhouse at 20 to 22°C, a light intensity of 20,000Lux, and a 16-h photoperiod. Finally, the fluorescence of eGFP was observed under a laser confocal fluorescence microscope at 48h post-infiltration.

### Verification of Transcription Activation by TaMYB29 Protein

The gene of full-length TaMYB29_1–261_ protein, its N-terminal region TaMYB29_1–116_, and C-terminal region TaMYB29_117–261_ were amplified using three pairs of primers containing *Nde*I and *Eco*RI restriction sites, namely, M29-BD-F/R, M29-BD-F/R_116_, and M29-BD-F_117_/R, respectively. The PCR products were purified and linked to the pGBKT7 vectors linearized by restriction endonucleases. The three pGBKT7-*TaMYB29* recombinant plasmids were directly transformed into DH5α *E. coli* strain and amplified. Subsequently, the pGBKT7-*TaMYB29* recombinant plasmids and the pGBKT7 plasmid (negative control) were introduced into the AH109 yeast strain containing the *HIS3*, *ADE2*, *MEL1*, and *lacZ* reporter genes. The transformed yeast strains were plated on a medium lacking tryptophan (SD/-Trp) and incubated at 30°C for 2 to 3days. The positive clones on the Trp-deficient media were selected and transferred into the yeast peptone dextrose adenine (YPDA) liquid media for culturing. Next, the yeast solutions were diluted to OD_600_ of 1, 10^−1^ 10^−2^, 10^−3^, and 10^−4^. Lastly, these yeast solutions were inoculated into a medium lacking adenine, histidine, and tryptophan (SD-His/-Ade/-Trp) as well as a medium lacking the above three amino acids (SD-His/-Ade/-Trp) and supplemented with X-α-D-galactoside (X-α-Gal). These cultures were incubated at 30°C for 3 to 4days.

### Transient Expression of *TaMYB29* in *Nicotiana benthamiana*

The *TaMYB29* gene was amplified using a pair of primers (PVX-M29-F/R) containing *Sma*I and *Not*I restriction enzyme sites. The PCR product was subcloned into the pMD18-T simple vector. Afterward, the recombinant plasmid was amplified in DH5α *E. coli* strain and digested by *Sma*I and *Not*I restriction enzymes. Subsequently, the gene digestion product was inserted into the PVX vector which carries a 3×HA tag and was linearized by the same restriction enzymes. The PVX-*TaMYB29* construct was transformed into the *Agrobacterium* strain GV3101 (pSoup-p19). Strains were grown in the Luria–Bertani (LB) medium containing 50mg/ml rifampicin and 25mg/ml kanamycin at 28°C for 24h. The bacteria were harvested by centrifugation and resuspended in the acetosyringone (AS) buffer containing 10mm MES (pH 5.6), 10mm MgCl_2_, and 150μm acetosyringone after being washed with 10mm MgCl_2_. Strains carrying different recombinant vectors were adjusted to a density of 0.6 at 600nm (OD_600_) with AS buffer and incubated for 2h in the dark at room temperature before leaf infiltration. These leaves were sampled at 1, 2, and 3days post-infiltration (dpi) and stained with 3,3′-diaminobenzidine (DAB) or trypan blue (Bai et al., 2012). The PVX-*GFP* construct was used as a control and treated in the same manner. These experiments were repeated at least three times and got the same result.

### BSMV-VIGS-Mediated *TaMYB29* Gene Silencing

A conserved region of about 200bp was selected from the cloned *TaMYB29* sequence to design the primers (M29-vigs-F/R) for silencing the fragments. The PCR product was linked to the BSMV:γ modified vector to construct the BSMV:γ-*TaMYB29* recombinant plasmid as previously described (Wang et al., 2020a). Next, the positive plasmid was amplified in DH5α *E. coli* strain. A construct carrying only the BSMV genome was used as a negative control and named BSMV:00. A construct carrying a 183-bp *phytoene desaturase* (*PDS*) gene was used as a positive control and named BSMV:*PDS*. The recombinant γ virus vector linearized by *BssH*II and RNAα, RNAγ linearized with *Mlu*I, and RNAβ with *Spe*I were transcribed *in vitro* using the mMessage mMachine T7 *in vitro* transcription kit (Ambion, Austin, TX, United States) following the manufacturer’s protocol. The transcription products of BSMV:α, β, γ (or γ-target) were diluted thrice with diethylpyrocarbonate (DEPC) water and mixed in a ratio of 1:1:1. Next, 70μl of FES buffer which is a kind of inoculation buffer containing a wounding agent was added to every 30μl of the mixture. The second leaves of AvS and AvS+*Yr10* wheat seedlings were infected with BSMV using the method described by Holzberg et al. (2002). The BSMV-infected wheat seedlings were maintained in a 16h/8h photoperiod at 25°C. In addition, the seedlings were mock inoculated with FES buffer devoid of BSMV transcripts. At 10 to 14days after the virus inoculation, photobleaching was observed on BSMV:PDS seedlings. The third and fourth leaves were further inoculated with uredospores of *Pst* pathotype CYR32. Next, these plants were directly maintained in a growth chamber for 24h in the dark with 100% relative humidity at 15°C. The leaves were sampled at 0, 24, 48, and 120hpi for histological changes and analyzed by qRT-PCR.

### Histological Study of Fungal Growth and Host Response

Wheat leaves sampled at 0, 24, 48, and 120hpi in control and silenced plants were observed for ROS accumulation by DAB staining and HR response areas because of the auto-fluorescence of necrotic cells using an Olympus BX-51 fluorescence microscope (Olympus Corp. Tokyo, Japan). Then, the leaf segments were stained with wheat germ agglutinin (WGA) and observed for the infection areas and hyphae development of *Pst* as previously described (Wang et al., 2017). At least 30 infection sites were examined on each of five randomly selected leaf segments per treatment. Infection sites with substomatal vesicles were considered successfully penetrated. Standard deviations were calculated and Tukey’s test was performed for statistical analysis using the SPSS software (SPSS, Inc. Chicago, United States).

## Results

### Sequence Analyses of *TaMYB29* cDNA and Protein

A full-length cDNA fragment was successfully isolated due to its high expression under incompatible interaction of wheat cv. AvS+*Yr10* infected with avirulent *Pst* CYR32. The cDNA sequence displayed 100% identity with the referred *TaMYB29* sequence in the NCBI GenBank (accession no. JF951912.1). In addition, the ORF was cloned using a pair of primers to create a 786-bp fragment. The International Wheat Genomic Sequence Consortium (IWGSC) revealed homologs of *TaMYB29* located on chromosomes 5A, 5B, and 5D in wheat cv. Chinese Spring. The alignment results of these nucleotide sequences also showed a high similarity ranging from 96 to 99%, referred to as *TaMYB29-5A*, *TaMYB29-5B*, and *TaMYB29-5D* (Supplementary Figure S1). The *TaMYB29* sequence of AvS+*Yr10* displayed the highest similarity to the nucleotide sequence of the Chinese Spring *TaMYB29-5B*.

**Figure 1:**
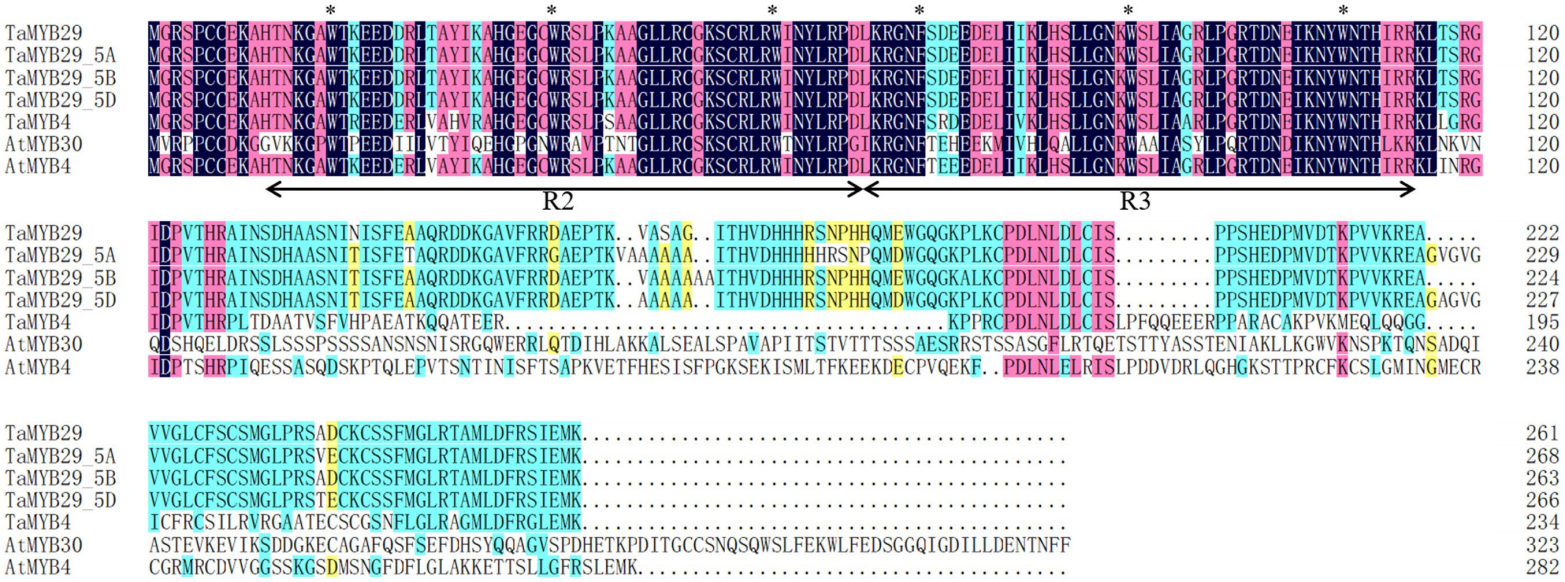
Alignment of the amino acid sequence of TaMYB29 with its homologous sequences. The sequences were aligned with DNAMAN. The identical residues are shaded. The black arrow line shows the MYB-binding domain. The asterisks (∗) show typical R2R3-MYB protein contains three regularly spaced tryptophan (W) residues and the first tryptophan residue was replaced by phenylalanine (F) in the R3 repeats.

The deduced protein of *TaMYB29* had 261 amino acids, with a predicted molecular weight of 29.4 kD and an isoelectric point (pI value) of 9.05. Alignments of all wheat alleles of TaMYB29 with wheat TaMYB4 (AEG64799.1), *Arabidopsis* AtMYB30 (AEE77505.1), and *Arabidopsis* AtMYB4 (AEE86955.1) revealed two highly conserved regions R2 and R3, which contained three uniformly spaced tryptophan residues responsible for the interaction between the MYB protein and specific DNA sequences (Figure 1). The second and third tryptophan residues were conserved in all R2R3-MYB protein members, except in R3 repeats, where the first tryptophan was replaced by phenylalanine (Zhang et al., 2012a). These results revealed highly conserved amino acid sequences in these MYB proteins, indicating their similar functions in mediating stress response.

### Subcellular Localization of TaMYB29 Protein

**Figure 2:**
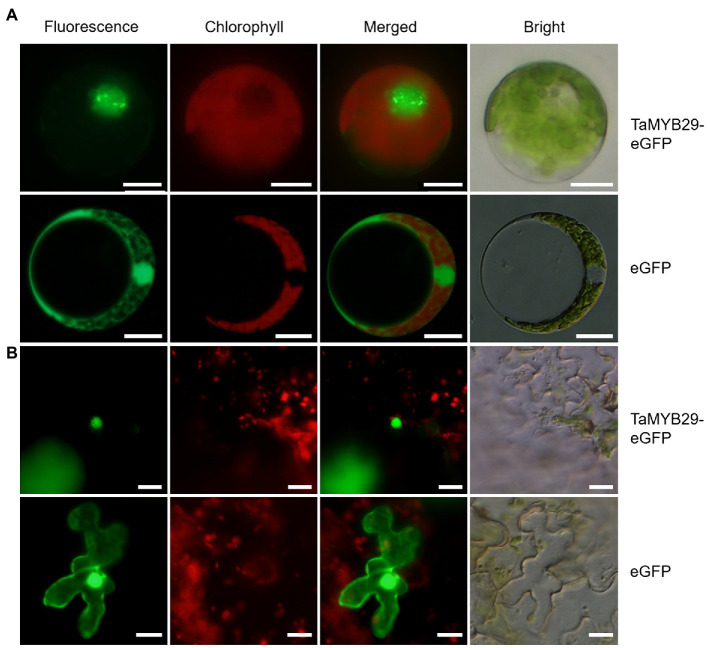
Subcellular localization of TaMYB29 protein. **(A)** TaMYB29-eGFP fusion protein and enhanced green fluorescence protein (eGFP) were expressed in wheat protoplasts; **(B)** TaMYB29-eGFP fusion protein and eGFP protein were expressed in the leaves of *Nicotiana benthamiana*. Bars=20μm.

To study the subcellular localization of TaMYB29 in plants, the target gene fused with the eGFP vector and only eGFP vector (control) was separately transformed into wheat protoplasts. To avoid the interference of chlorophyll, its distribution was studied individually. After analyzing the fluorescence microscopy observations, the exact localization of TaMYB29-eGFP fusion protein was found to be the nucleus of wheat protoplast, whereas green signals of only eGFP were observed both in the cytoplasm and the nucleus (Figure 2A). We subsequently used the leaves of *N. benthamiana* as the transformed material and repeated the subcellular localization experiment and obtained the same results (Figure 2B). Therefore, we conclude that TaMYB29 is a nuclear protein.

### Transcriptional Activity of TaMYB29 Protein

**Figure 3:**
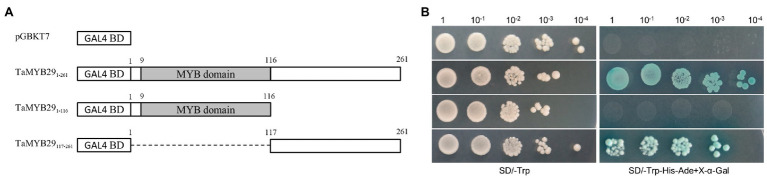
Transcriptional activation activity of the TaMYB29 proteins. **(A)** Constructs of pGBKT7-TaMYB29_1–261_ (full-length) and pGBKT7-TaMYB29_1–116_ (N-terminal region), pGBKT7-TaMYB29_117–261_ (C-terminal region), and pGBKT7 as a control were separately transformed into the yeast strain AH109. **(B)** The diluted yeast solutions were dropped on the SD/-Trp and SD/-Trp-His-Ade+X-α-Gal media, respectively; then, the cultures were incubated at 30°C for 3–4days.

To identify whether the TaMYB29 protein is a transcriptional activator or repressor, pGBKT7-TaMYB29_1–261_ and its two truncated protein plasmids pGBKT7-TaMYB29_1–116_ and pGBKT7-TaMYB29_117–261_ were transformed into the AH109 yeast strain. In addition, the pGBKT7 plasmid (negative control) was transformed simultaneously. All transformants grew well on a selective synthetic defined (SD) medium lacking tryptophan (SD/-Trp); however, only pGBKT7-TaMYB29_1–261_ and pGBKT7-TaMYB29_117–261_ yeast strains showed growth on the selective medium without tryptophan, histidine, and adenine (SD-Trp/-His/-Ade). Furthermore, the colonies turned blue on the same medium supplemented with X-α-Gal (SD-Trp/-His/-Ade+X-α-Gal; Figure 3). Therefore, we conclude that TaMYB29 functions as a transcriptional activator. What is more, the transactivation domain of TaMYB29 is located in the C-terminal region.

### Expression Profile of *TaMYB29* in Response to Exogenous Hormones

**Figure 4:**
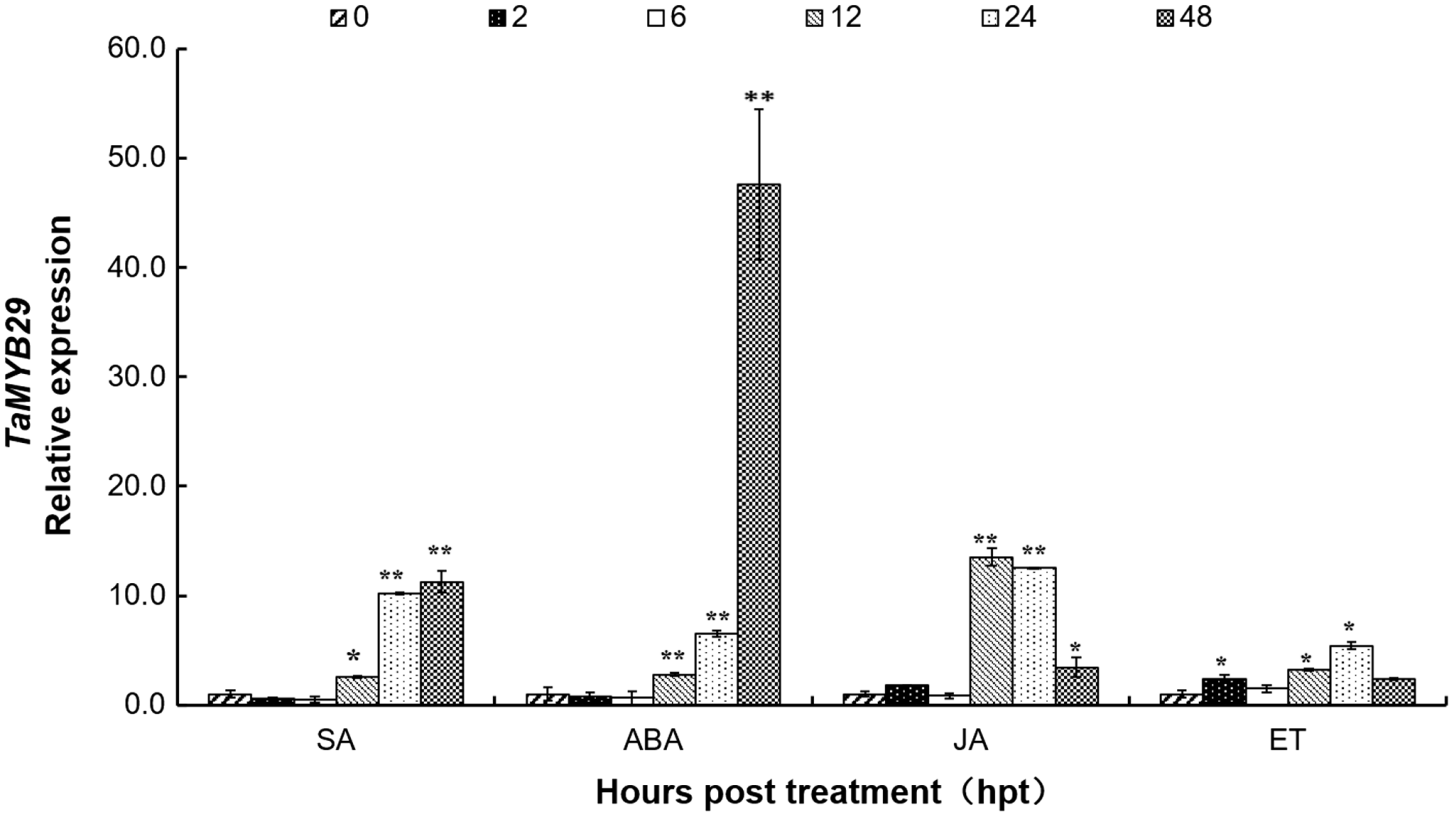
The expression profiles of *TaMYB29* in AvS+*Yr10* wheat leaves in response to exogenous hormones. The wheat leaves were sprayed with SA (salicylic acid), ABA (abscisic acid), JA (methyl jasmonate), and ET (ethylene), respectively and were sampled at 0, 2, 6, 12, 24, and 48h post-treatment (hpt). Three independent biological replications were performed. Error bars represent standard deviation among three biological replications. Student’s *t*-tests were used for the statistical analyses and the asterisks (∗) and (∗∗) indicate a significant difference between that time point and 0hpt with a value of *p*<0.05 and 0.01, respectively.

To understand the regulation of *TaMYB29* by plant hormones, the AvS+*Yr10* wheat seedling leaves were treated with four different exogenous hormones (SA, ABA, JA, and ET) at the two-leaf stage. The *TaMYB29* transcript levels were detected by qRT-PCR at six different time points (Figure 4). Regarding SA treatment, the relative expression of *TaMYB29* showed a 2.59-fold increase at 12h and a 10-fold increase at 24h. Eventually, the increase at 48h was 11 times more than that in the control at 0h. The *TaMYB29* expression of leaves treated with ABA showed a similar increasing trend. However, the increase was considerably high at 48h, 50 times as high as that in the control. With respect to JA treatment, the highest level was measured at 12h, with upregulation recorded from 12h to 48h. The upregulation was also observed in ET-treated leaves, especially at 2h, 12h, and 24h. However, the expression peak of *TaMYB29* induced by JA or ET treatment was not as high as that detected with the ABA treatment. These results demonstrate that *TaMYB29* exert an effect in the plant through complex hormone signal pathways.

### SA Levels and Expression Profiles of *TaMYB29* and *TaPR1* in Response to *Pst*

**Figure 5:**
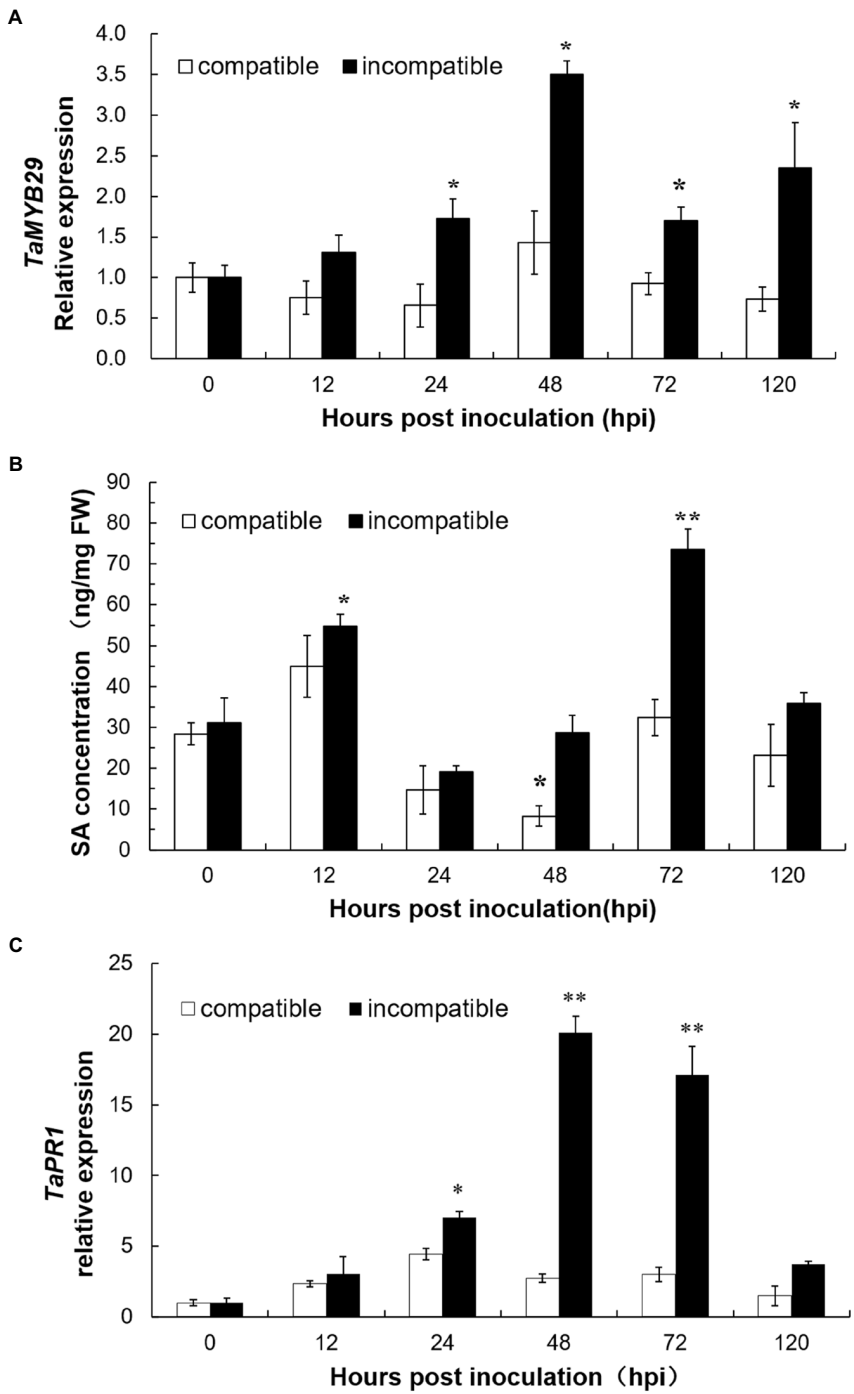
The expression profiles of *TaMYB29* **(A)** and *TaPR1* gene expression **(C)** and salicylic acid (SA) concentration **(B)** in AvS and AvS+*Yr10* wheat leaves during the time course of stripe rust infection. The leaves were sampled at 0, 12, 24, 48, 72, and 120h post-inoculation (hpi) with three biological experiments replicates. Error bars represent standard deviation among three biological replicates. Student’s *t*-tests were used to for the statistical analyses and the asterisks (∗) and (∗∗) indicate a significant difference between the particular time point and 0hpi with a value of *p*<0.05 and 0.01.

To study the expression profile of *TaMYB29* during the interaction between wheat and stripe rust pathogen, the relative expression of *TaMYB29* in the wheat leaves was measured by qRT-PCR at 0, 12, 24, 48, 72, and 120h in two different wheat cultivars post-inoculation with CYR32 (Figure 5A). Wheat AvocetS (AvS), which is susceptible to *Pst* pathotype CYR32, forms a compatible interaction, and its near-isogenic line AvS*Yr10*NIL (AvS+*Yr10*), carrying the *Yr10* resistance gene and highly resistant to CYR32, forms an incompatible interaction. The expression of *TaMYB29* in the leaves of AvS+*Yr10* plants was significantly upregulated at four different time points, except 12hpi in contrast to the 0hpi control and peaked at 48hpi, nearly 3.5 times more than that in the control. However, the expression of *TaMYB29* in the leaves of AvS plants showed an opposite trend, declining slightly until 24hpi at first. Although the transcript abundance increased slightly at 48hpi compared with 0hpi, it decreased to nearly half the level at 0hpi in the end at 120 hpi. The expression of *TaMYB29* in the incompatible interaction was always higher than that in the compatible interaction at five different time points. These results indicate that TaMYB29 functions during the resistance of wheat against *Pst* infection, especially in the incompatible interaction.

We measured the endogenous levels of SA in both compatible and incompatible interactions at six time points (Figure 5B). In the incompatible interaction, the first significant increase in the SA levels appeared at 12 hpi, earlier than the *TaMYB29* expression, and subsequently reduced at 24 and 48hpi, contrary to the observation in the *TaMYB29* expression compared with the 0hpi control. The highest SA levels were obtained at 72hpi. Next, the SA levels rapidly returned to the basal levels at 120hpi. The SA levels in the compatible interaction presented a similar trend as in the incompatible system although the levels were lower than those in the incompatible system at every time point.

Furthermore, the expression of the wheat *PR* gene *TaPR1* was measured by qRT-PCR (Figure 5C). *TaPR1* was upregulated in both interactions during pathogenesis. In the incompatible interaction, the expression of *TaPR1* significantly increased at 24hpi and reached the 20-fold increase at 48hpi, and maintained significantly high levels at 72hpi, which is considerably similar to the expression of *TaMYB29*. Next, it decreased at 120hpi, different from the expression of *TaMYB29*. In addition, the expression of *TaPR1* in the compatible interaction was slightly upregulated; however, the level was lower as compared with that in the incompatible interaction.

### TaMYB29-Induced ROS Accumulation and Pathogen-Independent Cell Death in *Nicotiana benthamiana* Leaves

**Figure 6:**
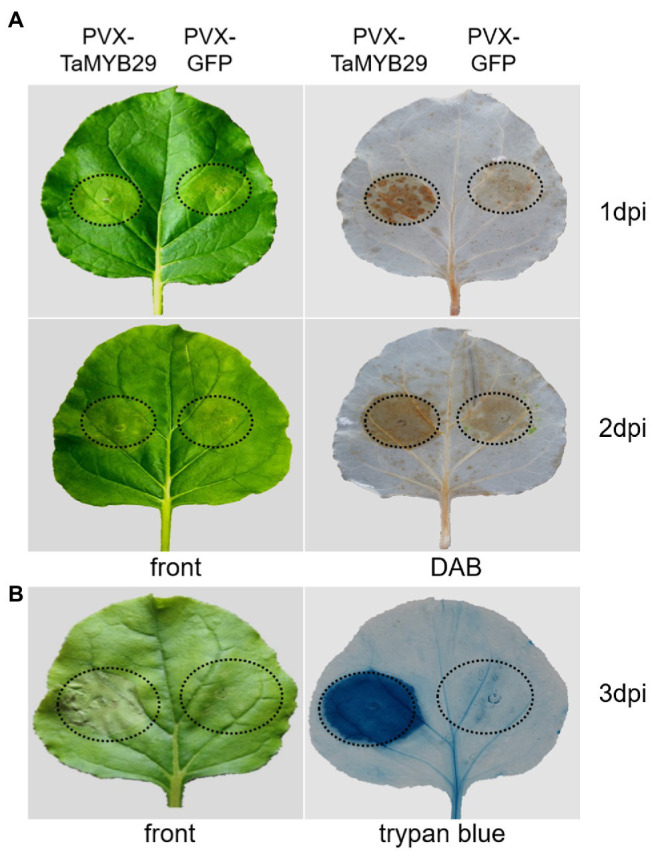
Expression of *TaMYB29*-induced ROS accumulation and pathogen-independent cell death in *N. benthamiana* leaves. **(A)** Symptoms of *N. benthamiana* leaf areas expressing *TaMYB29* or *GFP* gene from 1 to 2days post-infiltration (dpi). The left and right panels represent the front and after DAB staining, respectively. **(B)** Symptoms of *N. benthamiana* leaf areas expressing *TaMYB29* or *GFP* gene at 3 dpi. The left and right panels represent the front and after trypan blue staining, respectively. These experiments were repeated at least three times and got the same result.

The recognition of certain molecules released by pathogens triggers plant cell death and a subsequent defense response known as the HR response (Coll et al., 2011). To confirm whether *TaMYB29* can induce ROS and cell death, the HR symptoms were detected in *N. benthamiana* leaves transformed by PVX:*TaMYB29* and PVX:*GFP* constructs. After 1 to 2days post-infiltration (dpi), the accumulation of ROS was detected successfully with DAB staining (Figure 6A). The ROS accumulation in leaf areas infiltrated with PVX:*TaMYB29* was apparent in contrast to that in control PVX:*GFP*. Subsequently, apoptosis was observed using trypan blue staining to further investigate the effects of *TaMYB29* expression during ROS accumulation and pathogen-independent cell death (Figure 6B). The *TaMYB29*-induced cell death on *N. benthamiana* leaves was apparent. However, PVX:*GFP* control did not trigger cell death. The results showed that the expression of *TaMYB29* successfully led to ROS accumulation and cell death in contrast to the *GFP* control, indicating that *TaMYB29* played a crucial role in ROS accumulation and pathogen-independent cell death.

### *TaMYB29* Gene Silencing of Wheat Reduced the Resistance to Stripe Rust

**Figure 7:**
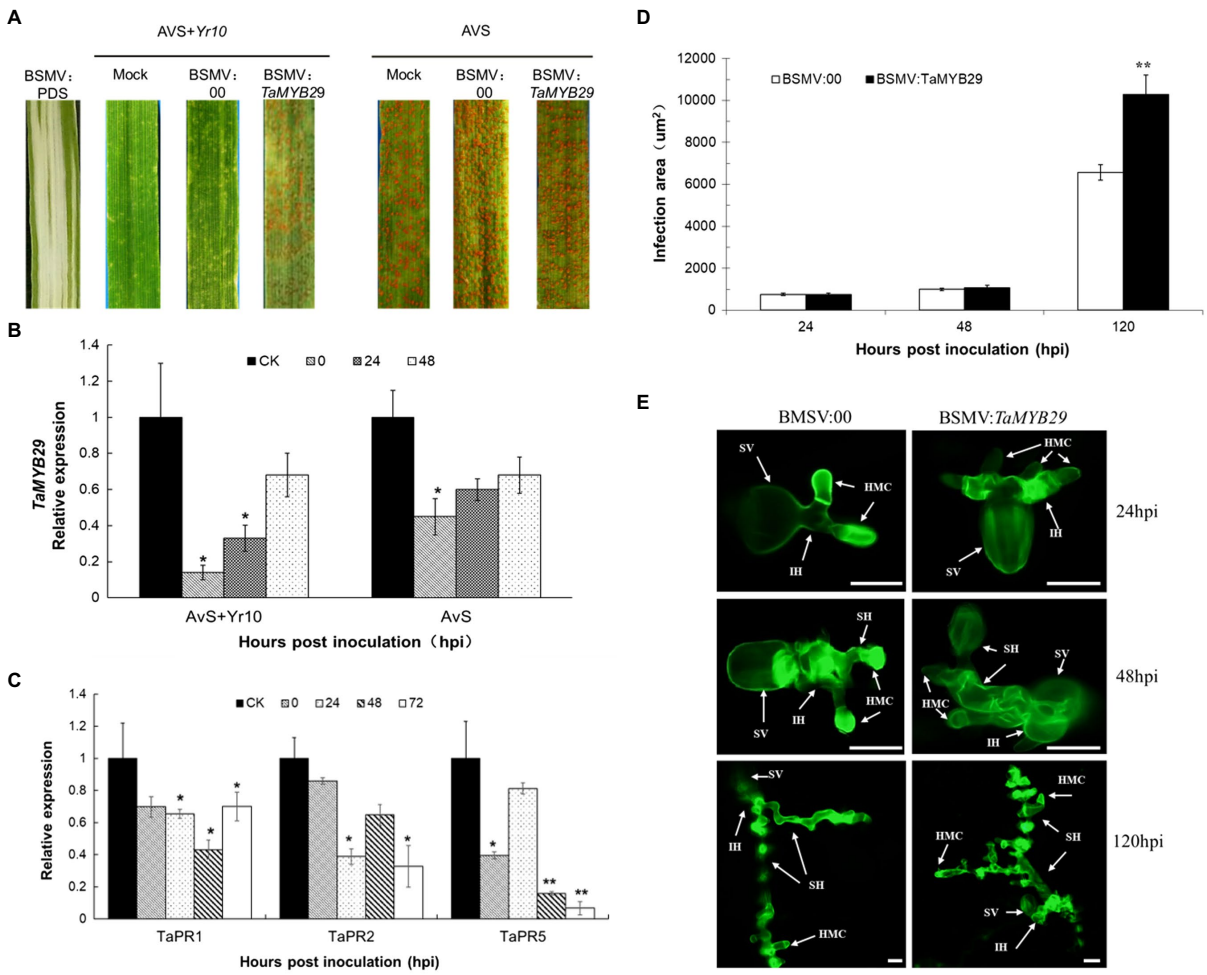
Functional analysis of the *TaMYB29* gene by the BSMV-VIGS assay. **(A)** Infection type of AvS and AvS+*Yr10* wheat leaves to CYR32 after inoculated with BSMV:00 or BSMV:*TaMYB29*. Stripe rust was inoculated 9days post-BSMV inoculation. Pictures were taken 15days post-rust inoculation. Mock: wheat leaves inoculated with FES buffer. BSMV:00 means only the BSMV genome; BSMV: *TaMYB29* means a 183-bp fragment of *TaMYB29* was inserted into the BSMV:γ genome. Photobleaching was evident on the leaves infected with BSMV:*PDS* but not on the mock-inoculated leaves. **(B)** Transcriptional level of *TaMYB29* in AvS and AvS+*Yr10* during the course of *Pst* infection at 0, 24, and 48hpi. CK: wheat leaves treated with BSMV:00. **(C)** Transcriptional level of pathogenesis-related gene *TaPR1*, *TaPR2*, and *TaPR5* in *TaMYB29* silenced AvS+*Yr10* wheat leaves after inoculated with *Pst*. Leaves were sampled at 0, 24 48, and 72hpi. Three independent biological replications were performed. Error bars represent standard deviation among three biological replicates. Student’s *t*-tests were used for the statistical analyses and the asterisks (∗) and (∗∗) indicate a significant difference between that time point and 0hpi with a value of *p*<0.05 and 0.01, respectively. **(D,E)** Infection area **(D)** and hyphae development of *Pst* **(E)** in *TaMYB29* knockdown AvS+*Yr10* wheat leaves after infected with CYR32. Wheat leaves were sampled at 24, 48, and 120hpi and observed microscopically after stained with WGA. SV, substomatal vesicle; IH, initial hyphae; HMC, haustorial mother cell; and SH, secondary hyphae. Bars=20μm.

To study the effects of the *TaMYB29* gene during the interaction between wheat and *Pst*, the target gene was silenced using the barley stripe mosaic virus-induced gene silencing (BSMV-VIGS) assay. At the beginning of the knockdown experiment, the *PDS* gene was used to ensure that the VIGS system worked normally and correctly. The photobleaching occurred on BSMV:*PDS* leaves, proving the effectiveness of the VIGS system (Figure 7A).

AvS and AvS+*Yr10* wheat seedlings infected with FES buffer (mock), BSMV:00, and BSMV:*TaMYB29* were further inoculated with uredospores of CYR32. Infection types were assessed based on McNeal measurement (Mcneal et al., 1971) to evaluate the differences between the phenotypes of mock, BSMV:00, and BSMV:*TaMYB29*. AvS+*Yr10* displayed a resistant response after inoculation with CYR32 on the mock and BSMV:00 controls, characterized by a large necrosis area at the infection site. While a small number of fungal spores appeared on *TaMYB29*-silenced leaves. These results indicated that the resistance of wheat AvS+*Yr10* with silenced *TaMYB29* gene resulted in a significant decline from highly resistant to moderately susceptible. There was no susceptible phenotype change in *TaMYB29*-silenced AvS wheat compared with the control mock and BSMV:00 (Figure 7A). The relative expression of *TaMYB29* was detected by qRT-PCR after *TaMYB29* gene silencing before *Pst* inoculations labeled as 0hpi, and 24hpi, and 48hpi during *Pst* infection. The results showed that the *TaMYB29* transcription level decreased in both AvS+*Yr10* and AvS, which was knocked down up to 86% in incompatible interaction and 55% under compatible interaction compared with BSMV:00 labeled as CK (control check) in Figure 7B.

Pathogenesis-related genes are vital for mounting disease resistance responses in plants. *TaPR1*, *TaPR2*, *and TaPR5* have been reported to be involved in systemic acquired resistance (SAR)—a type of plant immunization (Wang et al., 2020b). Therefore, we selected three *PR* genes as defense-related genes to investigate the effects of the knockdown of *TaMYB29* before and during *Pst* inoculation. The results showed that the decline of *TaPR1*, *TaPR2*, and *TaPR5* gene expression in BSMV:*TaMYB29* compared with BSMV:00 was observed at four different time points in the incompatible interaction (Figure 7C), proving that the silencing of *TaMYB29* reduced the wheat resistance to disease.

The infection area of *Pst* in *TaMYB29*-silenced AvS+*Yr10* wheat leaves was examined microscopically after inoculation with CYR32. The results showed that the total infection area in *TaMYB29*-silenced leaves was similar to that in BSMV:00 control until 48hpi. However, significant differences between the two groups were observed at 120hpi (Figures 7D,E). Furthermore, the hyphae development of *Pst* in BSMV:*TaMYB29* was stronger than that in BSMV:00, especially at 120hpi (Figures 7D,E).

In conclusion, *TaMYB29* knockdown effectively reduced wheat resistance to stripe rust by downregulating the expression of *PR* genes and promoting the development of hyphae.

### *TaMYB29* Was Involved in ROS Accumulation and Hypersensitive Response in Wheat

**Figure 8:**
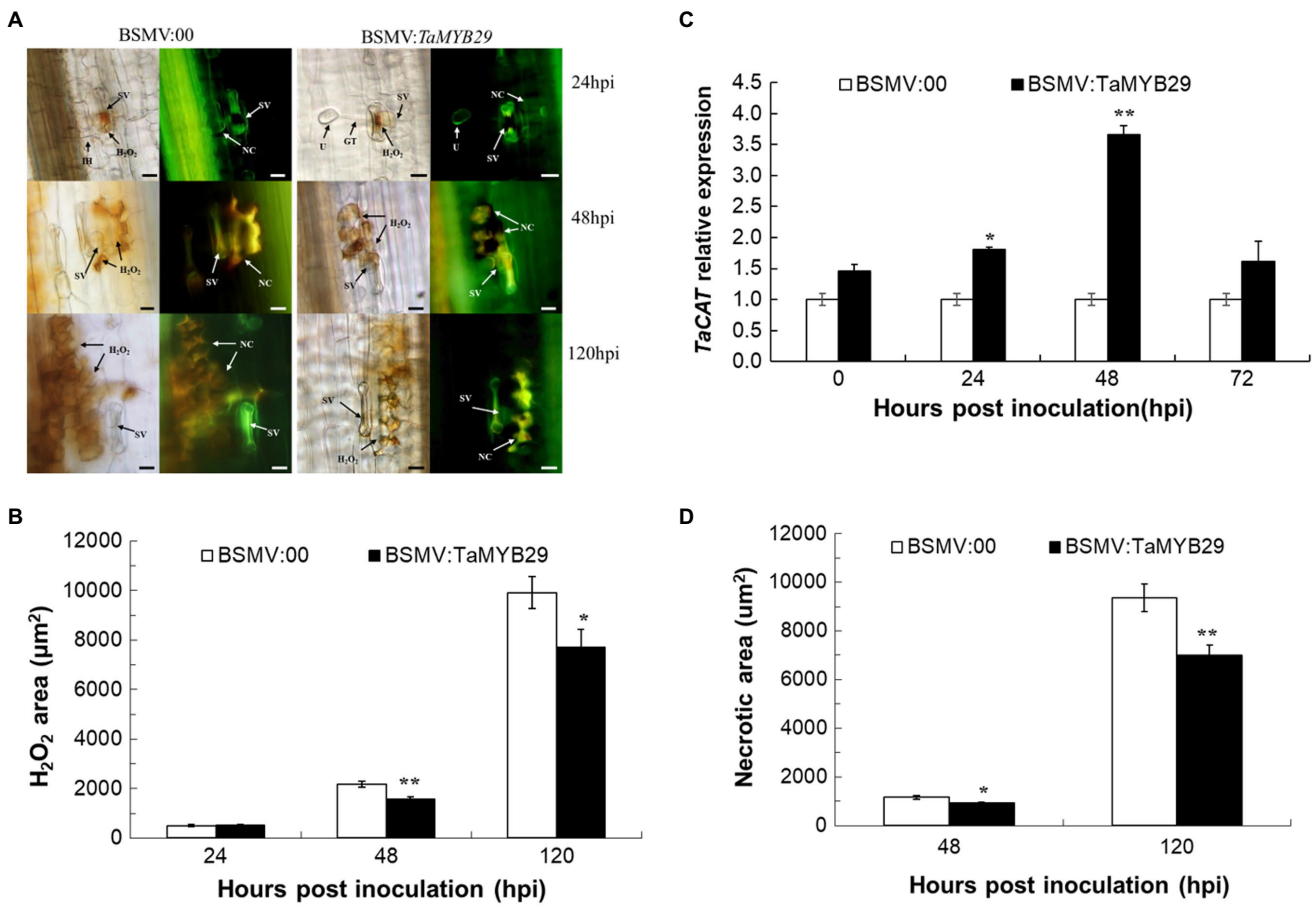
Reactive oxygen accumulation and related gene expression and hypersensitive response in the *TaMYB29* knockdown AvS+*Yr10* wheat leaves. **(A)** The reactive oxygen species and cell necrosis in wheat leaves in response to *Pst* observed under a fluorescence microscope. **(B)** The reactive oxygen species areas were stained with DAB and measured microscopically at 24, 48, and 120hpi. H_2_O_2_ accumulation was significantly reduced in *TaMYB29* silenced leaves compared to the control. **(C)** Transcriptional level of ROS-related gene *TaCAT* in AvS+*Yr10* after *TaMYB29*-scilencing at four time points relative to the expression in BSMV:00. **(D)** Cell death was measured microscopically because of the auto-fluorescence of necrotic cells at 48 and 120hpi. The area of green auto-fluorescence reduced in *TaMYB29*-silenced wheat plant. Pictures were taken under an epifluorescence or light microscopy at 24, 48, and 120hpi, respectively. BSMV:00 is the control group and BSMV:*TaMYB29* is the experimental group in which *TaMYB29* gene was silenced. U, urediniospore; GT, germ tube; NC, necrotic cell; SV, substomatal vesicle; IH, initial hyphae; HMC, haustorial mother cell; and SH, secondary hyphae. Bars=20μm. Error bars represent standard deviation among three biological replications. Student’s *t*-tests were used to for the statistical analyses and the asterisks (∗) and (∗∗) indicate a significant difference between particular hpi and controls with a value of *p*<0.05 and 0.01, respectively.

We observed the accumulation of ROS and cell necrosis of AvS+*Yr10* wheat leaves upon pathogen challenge. The production of H_2_O_2_ and the necrotic area in BSMV:*TaMYB29* leaves were significantly less than those in BSMV:00 at 48hpi and 120hpi, creating a gap of about 2,000μm^2^ at 120hpi (Figures 8A,B,D). In addition, the expression patterns of *TaCAT* (X94352) that can eliminate H_2_O_2_ as a catalase gene in wheat were detected by qRT-PCR (Coleto et al., 2021). The results showed that the expression of *TaCAT* was significantly upregulated in *TaMYB29*-silenced leaves at 24hpi, peaked at 48hpi as compared with control non-*TaMYB29*-silenced leaves (Figure 8C), implying that a high expression of *TaCAT* reduced the ROS accumulation in *TaMYB29*-silenced wheat leaves. We hypothesized that the silencing of the *TaMYB29* gene positively regulated the expression of the *TaCAT* gene, eventually decreasing the accumulation of ROS and partially eliminating the HR response in wheat mesophyll cells, thereby weakening the resistance of wheat to stripe rust.

## Discussion

We successfully cloned a relatively highly expressed *MYB* gene in response to the *Pst* attack in AvS+*Yr10* wheat leaves. The cDNA sequence showed the same to the *TaMYB29* gene (JF951912.1) searched using the NCBI GenBank database. Three homologous *TaMYB29* genes obtained from the wheat genome database maintained by the IWGSC were located to wheat chromosomes 5A, 5B, and 5D separately. Multiple sequence alignment revealed that the three genes shared 96 to 99% similarity, implying that their co-silencing effectively represented the function of *TaMYB29* genes in wheat. Therefore, we knocked down and measured the expression of three homologs simultaneously and used *TaMYB29* to represent their functions. More in-depth research is required to determine the specific functions of each of the three homologs.

The *MYB* gene superfamily participates in several metabolic reactions in plants (Jin and Martin, 1999). For example, these MYB proteins interact with DNA as transcript factors to regulate the signal network of the defense response. The majority of *MYB* genes belong to the R2R3-MYB superfamily; proteins in this superfamily have R2 and R3 imperfect tandem repeats DNA-binding domains. A comparison of amino acid sequences with the other three MYB family genes of *Arabidopsis* and wheat—all of which function to resist abiotic and biotic stresses—revealed that TaMYB29 also included R2 and R3 conserved domains, suggesting its DNA-binding ability as a transcription factor (Figure 1). Its subcellular localization analysis revealed that TaMYB29 is a nuclear protein (Figure 2). Transcriptional activation assays in yeast verified the transcriptional activity of TaMYB29 and the necessity of C-terminal 117–261 amino acids for transactivation (Figure 3). Based on these results, we speculated that the *TaMYB29* gene functions are similar to those of other resistance-related *MYB* genes previously reported as transcription factors (Morse et al., 2009; Katiyar et al., 2012; Jin et al., 2014).

The genes belonging to the *MYB* superfamily have been implicated in plant resistance against abiotic and biotic stresses (Saha et al., 2016). The expression of the *AtMYB30* gene increased during incompatible interactions between *Arabidopsis* and bacterial pathogens and drought stress (Vailleau et al., 2002). The expression of *AtMYB44* was upregulated following pathogen infection and treatment with defense-related SA phytohormones (Zou et al., 2013). Similarly, the expression of *TiMYB2R-1* was significantly induced following *G. graminis* infection (Liu et al., 2013). The analysis of the expression patterns of 60 wheat *TaMYB* genes under different stress conditions revealed that 32 of them responded to these different stresses (Zhang et al., 2012a). Among these, *TaMYB29* was induced by both high salt and exogenous ABA (Zhang et al., 2012a). We also found that *TaMYB29* was induced by exogenous ABA, a finding same as that of Zhang’s study (Figure 4). In addition, we found that the transcription of *TaMYB29* was significantly induced and reached the highest level at 48h post-rust inoculation in the incompatible system (Figure 5A), implicating the involvement of *TaMYB29* in wheat defense response to biotic and abiotic stresses.

Plant hormones are synthesized *de novo* and serve as signal molecules between plants and other surrounding organisms. These are usually conserved in the plant kingdom and can regulate diverse processes including plant growth and development, and responses to abiotic and biotic stresses (Nambara and Wees, 2021). Salicylic acid is a key regulator of plant defense response against living parasitic fungi and the acquisition of SAR pathways. The primary underlying mechanism of SA is to activate the high expression of a series of transcription factors, including DNA-binding proteins containing conserved MYB domains. For example, AtMYB30, AtMYB44, and AtMYB96 participate in the resistance of *Arabidopsis* to *Pseudomonas syringae* pv. *tomato* strain DC3000 *via* the SA signaling pathway (Dong et al., 1999; Vailleau et al., 2002; Seo and Park, 2010). Wheat AvS+*Yr10* containing the stripe rust resistance gene *Yr10* was highly resistant to CYR32 in incompatible interaction. Real-time PCR revealed a rapid induction of *TaMYB29* as early as 12h post-SA treatment (hpt) in incompatible interaction. The highest 11-fold increase was recorded at 48hpt (Figure 4). In addition, it showed significantly increased endogenous SA levels in AvS+*Yr10* as early as 12hpi; the second peak appeared at 72hpi compared to the 0hpi control (Figure 5B). The expression of *TaMYB29* was significantly increased at 24hpi in the incompatible interaction, which was delayed by 12h compared to the SA (Figures 5A,B). These results suggested that *TaMYB29* functions downstream in the SA biosynthesis pathway to defend against *Pst* in the incompatible interaction.

Crosstalk between defense signaling pathways regulates the defense responses against different types of attackers (Kachroo and Kachroo, 2007; Vlot et al., 2009; Robert-Seilaniantz et al., 2011; Zou et al., 2013). Abscisic acid, an important plant hormone, is involved in plants’ response to environmental stresses, such as drought, high salinity, and extreme temperature and plant–pathogen interactions (Popko et al., 2010; Wang et al., 2017). Similarly, JA and ET are primarily involved in the interaction between plants with necrotrophic pathogens and insects as well as in the wounding process (Reymond et al., 2000). We observed that *TaMYB29* was significantly induced after treatment with SA, ABA, JA, and ET exogenous hormones; the highest induced expression was detected at 48h post-treatment with ABA (Figure 4). We previously found that endogenous SA concentration increased post-ABA and JA treatments and improved the wheat defense against stripe rust in Suwon11 (Wang et al., 2017). *AtMYB96*-mediated ABA signals induced pathogen resistance response by promoting SA biosynthesis in *Arabidopsis* (Seo and Park, 2010). *AtMYB44* is involved in both ET- and SA-mediated defense-related signaling pathways to regulate the plant defense response (Zou et al., 2013), implying crosstalks between SA, ABA, JA, and ET in wheat defense response to stripe rust. *TaPIMP1* contributes to biotic and abiotic stress resistance by regulating defense- and stress-related genes in the ABA–SA signaling pathways in wheat (Zhang et al., 2012b). SA- and JA-mediated plant defense signaling pathways have both synergistic and antagonistic effects. The SA pathway activated by biotrophic pathogen *P. syringae* strongly reduced JA-mediated defenses against the attack of necrotrophic pathogen *A. brassicicola* in *Arabidopsis* (Spoel et al., 2007). On the other hand, co-treatment with low concentrations of SA and JA resulted in a synergistic effect on the transcription of *PR1* in *Arabidopsis* explants (Mur et al., 2006). Exogenous SA and JA could induce the expression of *TaMYB29* in different time points, which indicates that TaMYB29 plays a role in the communication between SA- and JA-mediated resistance signaling pathways (Figure 4). Moreover, the transcript of *TaMYB29* is induced both following the treatment with SA, ABA, JA, and ET hormones, and during the defense response against stripe rust (Figures 4, 5B). In summary, these results indicate that *TaMYB29* functions in wheat defenses through a complex interaction, including the ABA–SA or JA/ET–SA signaling network.

Increasing evidence shows SA as a crucial signaling molecule in plant defense against pathogens. This defense response usually causes local cell necrosis, namely, HR response and SAR as well as *PR* gene expression (Durrant and Dong, 2004). Pathogenesis-related genes including *PR1*, *PR2*, and *PR5* are induced along with the defense response of plants to pathogens through SA signaling (Vlot et al., 2009). As an indicator gene of SA, the expression of the *PR1* gene was detected during compatible and incompatible interactions. The expression of *PR1* increased significantly at 24hpi, 48hpi, and 72hpi in the incompatible interaction like *TaMYB29*, which was 12h later than SA (Figure 5). Compared with the control, the silencing of *TaMYB29* reduced the expression of three *PR* genes (Figure 7C), the production of H_2_O_2_ (Figure 8B), and the HR areas (Figure 8D). These findings suggested that *TaMYB29* modulated the defense response in a *PR* gene expression-dependent manner through the signaling molecule SA. During plant defense responses, transcription factors mediate the regulation of the expression of plant host target genes, largely through the specific recognition of cis-promoter elements. Several putative MYB recognition sequences have been found in the promoter region of the *PR1* gene (Abe et al., 1997). The tobacco *MYB1* gene is induced during the HR response and SAR. The MYB1 protein binds to the MYB consensus-binding site in the tobacco *PR1-a* promoter *in vitro* (Yang and Klessig, 1996). In addition, AtMYB44 may regulate the *PR1* gene expression by binding to its promoter region (Zou et al., 2013). Combining the expression of *PR* genes and *TaMYB29* gene in plant defense against stripe rust with the results of *TaMYB29* gene silencing, we hypothesize that TaMYB29 binds to *PR* cis-elements to regulate its expression. However, we cannot exclude the possibility that TaMYB29 indirectly regulates *PR1* expression by regulating other target genes. These results suggest that AvS+*Yr10* defense against *P. striiformis* depends on the expression of *PR* genes *via* the SA signaling pathway.

*Yr10* gene encodes for a unique CC-NBS-LRR receptor in wheat cultivar Moro, which provides seedling or all-stage resistance (*R* gene-mediated resistance; Liu et al., 2014). The *R* gene-mediated resistance is characterized by rapid plant cell death at the infection sites, thereby hindering the fungus spread from the infection sites—a process known as plant HR response—and inducing SAR (Heath, 2000; Durrant and Dong, 2004; Ryals et al., 2013). Following the successful recognition of an avirulent gene from the pathogen by the *R* gene, ROS rapidly accumulate and are instantaneously released in a process termed “oxidative burst.” This is one of the earliest events of plant defense against pathogens. In our study, the transient overexpression of *TaMYB29* in tobacco caused a rapid ROS increase, consequently inducing cell death at the injection sites (Figure 6). Similarly, the overexpression of *AtMYB44* enhanced H_2_O_2_ accumulation and cell death *via* the SA signaling pathway (Zou et al., 2013). Downregulating the three homologs of *TaMYB29* in AvS+*Yr10* reduced the host resistance to the avirulent *Pst* strain CYR32, whereas no obvious phenotype difference between the *TaMYB29*-silenced AvS plants and controls was observed (Figure 7A), suggesting that the *TaMYB29* gene is indispensable for the resistance to *Pst* infection mediated by *Yr10*. In the incompatible interaction, only extremely little accumulation of H_2_O_2_ in both *TaMYB29*-silenced AvS+*Yr10* plants and the BSMV:00 control was observed at 24hpi. However, at 48hpi and 120hpi, the accumulation of H_2_O_2_ was reduced significantly in the *TaMYB29*-silenced mesophyll cells in contact with primary hyphae as compared with the control BSMV:00 (Figures 8A,B). We hypothesized that *TaMYB29* affected the accumulation of ROS, which was supported by the histological observation and measurement of the production of H_2_O_2_ after knocking down *TaMYB29* (Figures 8A,B). The accumulation of H_2_O_2_ was only observed in guard cells during the formation of appressorium at 24hpi (Figure 8A), which was about 12h later than that in Suwon11 inoculated with CYR23 (Wang et al., 2017). These results indicated that the wheat cultivars containing different *R* genes led to varying ROS production in response to different rust isolates. Catalase, a powerful antioxidant enzyme, is an H_2_O_2_ scavenger. We reported that a 3.66-fold expression of the catalase gene *TaCAT* was induced in *TaMYB29* knocked-down leaves infected with CYR32 compared with the BSMV:00 control at 48hpi (Figure 8C). We speculated the upregulated expression of *TaCAT* as one of the reasons for the reduced ROS accumulation. In addition, the infection area was significantly increased after knocking down *TaMYB29* at 120hpi in comparison with the BSMV:00 control wheat plants (Figures 7D,E). Altogether, *TaMYB29* activated the wheat defense response against *Pst* by modulating H_2_O_2_ accumulation. During the defense response against pathogens, *TaMYB29* is induced *via* the SA pathway, thus increasing the levels of ROS and the expression of *PR* genes, and eventually generating resistance of wheat containing the *Yr10* disease resistance gene to stripe rust. However, we cannot conclude whether *TaMYB29* is required in all *R* gene-mediated disease resistance pathways, which requires further research. The underlying mechanism of regulation of specific target genes by *TaMYB29* to induce the defense response as a transcription factor requires further research. Finally, the possible use of this gene for breeding disease-resistant plant varieties needs to be assessed.

## Conclusion

*TaMYB29* plays an indispensable role in the wheat response against stripe rust *via* regulating the crosstalk between various signaling pathways. Moreover, our results indicate that *TaMYB29* positively regulates the plant defense response against biological nutritional pathogens by enhancing H_2_O_2_ accumulation, *PR* gene expression, and cell death *via* the SA signaling pathway.

## Data Availability Statement

The original contributions presented in the study are included in the article/Supplementary Material, and further inquiries can be directed to the corresponding authors.

## Author Contributions

XW and ZK conceived the study. XW, XZ, XL, CL, and ZK advised on the experimental design and drafted the manuscript. XW, XZ, XL, QH, DG, CL, ZW, and JC performed experiments and did data analysis. XZ, XL, QH, DG, CL, ZW, and JC interpreted data. XW, XZ, XL, and ZK wrote the manuscript and other authors reviewed and revised the manuscript. All authors contributed to the article and approved the submitted version.

## Funding

This study was supported by the National Natural Science Foundation of China General Projects (No. 31501619 and No. 32172424), 2021 College Students’ innovation and entrepreneurship training program (S202110712787), Natural Science Foundation Research Project of Shaanxi Province (2021JM-095), Shaanxi Innovation Team Project (2018TD-004).

## Conflict of Interest

The authors declare that the research was conducted in the absence of any commercial or financial relationships that could be construed as a potential conflict of interest.

## Publisher’s Note

All claims expressed in this article are solely those of the authors and do not necessarily represent those of their affiliated organizations, or those of the publisher, the editors and the reviewers. Any product that may be evaluated in this article, or claim that may be made by its manufacturer, is not guaranteed or endorsed by the publisher.

## Footnotes

^1^ https://www.ncbi.nlm.nih.gov
