# Supplementary material for: TaMYB29: A Novel R2R3-MYB Transcription Factor Involved in Wheat Defense Against Stripe Rust
Source: Front Plant Sci. 2021 Nov 29;12:783388. doi: 10.3389/fpls.2021.783388 (PMC8666710; doi:10.3389/fpls.2021.783388)
Supplement: Supplementary file 1 [file Data_Sheet_1.docx]

Supplementary Material

Article title: TaMYB29: A novel R2R3-MYB transcription factor involved in wheat defense against stripe rust

Authors: Xiaoxu Zhu^1,2#^, Xiang Li^3#^, Qi He^1^, Dongxiao Guo^1^, Caiqi Liu^3^, Junying Cao^1^, Zhongyi Wu^1^, Zhensheng Kang^2*^ and Xiaojing Wang^1*^

The following supplementary materials are available for this article:


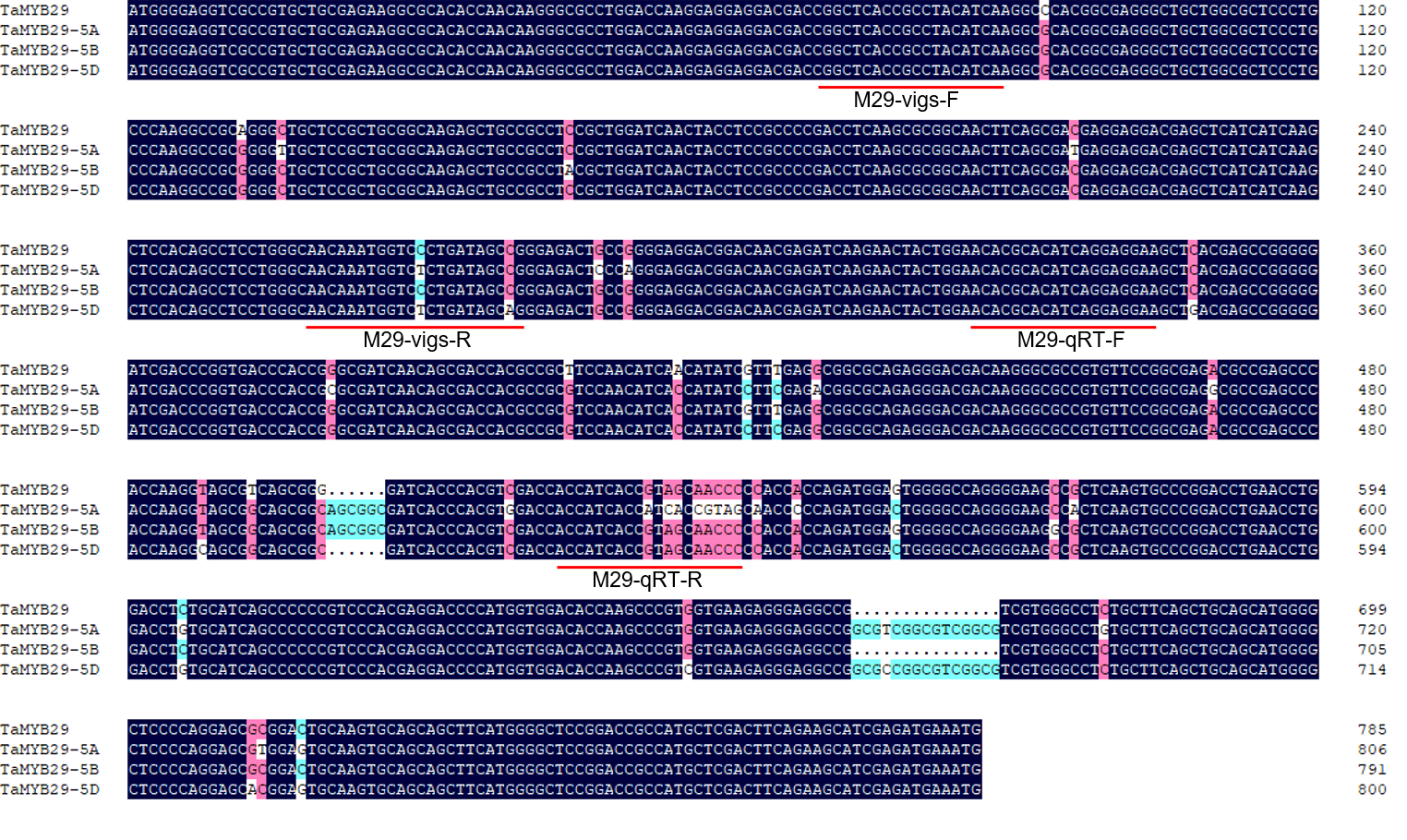


Fig. S1 Nucleotide sequences alignment between *TaMYB29* in AvS+*Yr10* and homologous sequences in Chinese spring. The primers for BSMV-VIGS and qRT-PCR were labeled by red lines.

Table S1 Sequences of the primers used in this study

| Primer name | Primer sequence (5’-3’) |
| --- | --- |
| M29-F | ATGGGGAGGTCGCCGTGC |
| M29-R | TTTCATCTCGATGCTTCTGAAGTCG |
| M29-qRT-F | ACACGCACATCAGGAGGAA |
| M29-qRT-R | GGGTTGCTACGGTGATGGT |
| M29-vigs-F | CGGCTCACCGCCTACATCA |
| M29-vigs-R | CGGCTATCAGGGACCATTTGTT |
| 163-M29-F | CCCAAGCTTATGGGGAGGTCGCCGTGC |
| 163-M29-R | CGGGATCCTTTCATCTCGATGCTTCTGAAGTCG |
| M29-getw-F | GGGGACAAGTTTGTACAAAAAAGCAGGCTTAATGGGGAGGTCGCCGTGC |
| M29-getw-R | GGGGACCACTTTGTACAAGAAAGCTGGGTATCATTTCATCTCGATGCTTCTGAAGTCG |
| M29-BD-F | TCAGAGGAGGACCTGCATATGATGGGGAGGTCGCCGTGC |
| M29-BD-R_116_ | TCGACGGATCCCCGGGAATTCGAGCTTCCTCCTGATGTGCGT |
| M29-BD-F_117_ | TCAGAGGAGGACCTGCATATGACGAGCCGGGGGATCGAC |
| M29-BD-R | TCGACGGATCCCCGGGAATTCTTTCATCTCGATGCTTCTGAAGTC |
| PVX-M29-F | TCCCCCGGGATGGGGAGGTCGCCGTGC |
| PVX-M29-R | ATTTGCGGCCGCTTTCATCTCGATGCTTCTGAAGTCG |
| TaPR1-qRT-F | GAGAATGCAGACGCCCAAGC |
| TaPR1-qRT-R | CTGGAGCTTGCAGTCGTTGATC |
| TaPR2-qRT-R | AGGATGTTGCTTCCATGTTTGCCG |
| TaPR2-qRT-R | AAGTAGATGCGCATGCCGTTGATG |
| TaPR5-qRT-R | CAAGCAGTGGTATCAACGCAGAG |
| TaPR5-qRT-R | GTGAAGCCACAGTTGTTCTTGATGTT |
| TaCAT-qRT-R | TGCCTGTGTTTTTTATCCGAGA |
| TaCAT-qRT-R | CTGCTGATTAAGGTGTAGGTGTTGA |
| actin-qRT-F | TGACCGTATGAGCAAGGAG |
| actin-qRT-R | CCAGACAACTCGCAACTTAG |
